# Risk Factors and Predictive Modeling of Occult Choledocholithiasis in Patients with Cholecystolithiasis

**DOI:** 10.1155/grp/9419737

**Published:** 2026-03-05

**Authors:** Ping Zhang, Long-jiang Chen, Dan-feng Liu

**Affiliations:** ^1^ Department of Surgery, The First People’s Hospital of WuHu, Wuhu, Anhui Province, China; ^2^ Wannan Medical College, Wuhu, Anhui Province, China, wnmc.edu.cn; ^3^ Department of Hepatobiliary Surgery, Affiliated Yijishan Hospital of Wannan Medical College, Wuhu, Anhui Province, China, wnmc.edu.cn; ^4^ Department of Hepatobiliary Surgery, Wuhu Hospital Affiliated to East China Normal University, Wuhu, Anhui Province, China

**Keywords:** asymptomatic choledocholithiasis, gallstone, occult common bile duct stone, prediction model, risk factors

## Abstract

**Background:**

Occult choledocholithiasis, if not diagnosed and treated in a timely manner, can have severe consequences. The purpose of this study is to construct a predictive model to assist in the diagnosis.

**Methods:**

A total of 988 case datasets were included. Data were analyzed using chi‐square tests and multivariate logistic regression. Ultimately, a predictive model for gallstones combined with occult choledocholithiasis was constructed.

**Results:**

Multivariate logistic regression analysis revealed that age, alanine aminotransferase (ALT), gamma‐glutamyl transferase (GGT), direct bilirubin (DBIL), location of gallstones, and ultrasonographic indication of common bile duct dilation are independent risk factors for gallstones combined with occult choledocholithiasis. A predictive model was constructed based on these factors: logit(P) = −5.109 + 2.007x1 + 1.175x2 + 3.479x3 + 1.412x4 + 2.199x5 + 2.473x6 (where x1–x6 represent age, location of gallstones, ultrasonographic indication of common bile duct dilation, ALT, GGT, DBIL, respectively). The model demonstrated a sensitivity of 0.839, specificity of 0.891, accuracy of 0.885, 95% CI of 0.913–0.967, and an AUC of 0.940.

**Conclusion:**

Age, ALT, GGT, DBIL, gallstone location, and sonographic common bile duct dilation constitute independent risk factors for gallstones with occult choledocholithiasis. The prediction model based on these indicators provides a valuable tool for the diagnosis of occult choledocholithiasis.

## 1. Introduction

Cholecystolithiasis, as a common biliary tract disease, has a prevalence rate of 2.3%–20% in adults [1, 2], with only 20% of patients presenting with clinical symptoms such as abdominal pain, while the remaining patients are mostly diagnosed with gallstones through ultrasound and other examinations [2, 3]. Approximately 10%–18% of patients with gallstones also have common bile duct stones (CBDS) [2, 4], which can lead to jaundice and even acute cholangitis, making their clinical diagnosis relatively straightforward. Some of these patients, who do not exhibit jaundice, abdominal pain, chills, or high fever, are referred to as having occult choledocholithiasis [5, 6]. Some literature also indicates that, in addition to the absence of clinical manifestations, patients with occult choledocholithiasis do not show abnormalities in related blood indicators such as liver enzymes, white blood cells, and C‐reactive protein [7–10]. In this study, occult choledocholithiasis specifically refers to the absence of related clinical manifestations (jaundice, chills, high fever, etc.), regardless of whether blood indicators are abnormal or not. Currently, the diagnostic protocols for gallstones all recommend routine preoperative abdominal US, and usually do not perform magnetic resonance cholangiopancreatography (MRCP) [11, 12]. US has a lower diagnostic rate for occult choledocholithiasis and may miss some patients, and some guidelines and researchers recommend or choose MRCP or endoscopic ultrasonography (EUS) as a diagnostic plan for occult choledocholithiasis [9, 13]. However, some hospitals may lack the technical or equipment capabilities, leading to potential missed diagnoses of occult choledocholithiasis. If occult choledocholithiasis is not diagnosed and treated in time, it can induce post‐cholecystectomy unexplained abdominal pain, or severe complications such as biliary pancreatitis or acute cholangitis, which can be life‐threatening. Therefore, accurately determining whether patients with gallstones have combined occult choledocholithiasis before surgery is particularly important for formulating treatment plans. This study retrospectively analyzed the clinical data of patients with gallstones combined with occult choledocholithiasis and conducted statistical analysis of their risk factors, thereby constructing a predictive model to provide a certain reference for the clinical diagnosis of gallstones combined with occult choledocholithiasis.

## 2. Materials and Methods

### 2.1. Study Subjects

A retrospective case–control study was conducted, strictly adhering to predefined inclusion and exclusion criteria, to collect clinical data from patients with gallstones who underwent surgical treatment at Wuhu Second People’s Hospital between January 2020 and June 2024.

Patients were included based on the following criteria: (1) Abdominal color Doppler US suggesting gallstones; (2) Availability of complete preoperative laboratory tests including blood routine, biochemical profile, infectious disease markers, and serum markers; (3) Performance of at least one of the following examinations: CT, MRCP, EUS, or Endoscopic Retrograde Cholangiopancreatography (ERCP).

The exclusion criteria were the following: (1) Physical examination at admission revealing positive signs of choledocholithiasis or cholecystitis such as chills or jaundice; (2) Abdominal color Doppler US suggesting choledocholithiasis; (3) History of alcoholic liver disease, hepatitis, primary sclerosing cholangitis, or other conditions affecting liver function; (4) History of biliary pancreatitis or upper abdominal surgery; (5) Diagnosis of malignant tumor.

Notably, patients who developed postoperative liver function abnormalities or biliary dilation were specifically excluded from this study. These abnormalities could potentially result from the surgical procedure itself (such as common bile duct exploration) or postoperative complications (including edema or retained stones), which contradicts the fundamental definition of “occult” choledocholithiasis—the absence of preoperative imaging evidence. This exclusion ensures homogeneity of the study population and maintains the purity of outcome definitions.

The diagnostic criteria for choledocholithiasis were as follows: (1) Confirmation through common bile duct exploration, or (2) Confirmation through ERCP.

### 2.2. Data Collection

The collected clinical data comprised the following: (1) general medical records, including name, gender, and age; (2) laboratory test results, including alanine aminotransferase (ALT), aspartate aminotransferase (AST), gamma‐glutamyl transpeptidase (GGT), alkaline phosphatase (ALP), total bilirubin (TBIL), direct bilirubin (DBIL), high‐density lipoprotein (HDL), low‐density lipoprotein (LDL), total cholesterol (TCHO), triglycerides (TG), amylase (AMY), and lactate dehydrogenase (LDH); and (3) imaging characteristics, including gallstone size, location, number, and the ultrasonographic indication of common bile duct dilation (US‐CBD dilation).

### 2.3. Statistical Methods

All statistical analyses were performed using *Rstudio* software. The collected clinical data were randomly divided into a training set and a validation set at a 7:3 ratio. Categorical variables in the training set were analyzed using the chi‐square test, and a multivariate logistic regression analysis was conducted to develop a predictive model for “gallstones combined with occult choledocholithiasis.” Internal validation was carried out via Bootstrap resampling, during which the optimism of the model’s AUC was calculated and corrected. Decision curve analysis (DCA) and receiver operating characteristic (ROC) curves were plotted for both the model and individual independent indicators. The model was further validated in the validation set to comprehensively evaluate its predictive performance. A *p* value < 0.05 was considered statistically significant.

## 3. Results

### 3.1. General Data

A total of 988 patients were included, with 344 males and 644 females. Patients diagnosed with simple gallstones numbered 865 (285 males, 580 females, average age 51 years), accounting for 87.6%; patients with combined occult choledocholithiasis numbered 123 (64 males, 59 females, average age 62 years), accounting for 12.4%. All patients underwent routine abdominal color Doppler US before surgery, performed by physicians with more than 10 years of experience in abdominal US at our hospital. Patients with simple gallstones underwent laparoscopic cholecystectomy (LC), while those with combined occult choledocholithiasis underwent open cholecystectomy with common bile duct exploration and stone removal or LC + LCBD or LC + ERCP.

### 3.2. Data Grouping

The data were randomly divided into a training set of 693 patients (244 males, 449 females) and a validation set. In the training set, there were 606 patients with simple gallstones (201 males, 405 females, average age 51 years) accounting for 87.4%, and 87 patients with combined occult choledocholithiasis (43 males, 44 females, average age 62 years) accounting for 12.6% (Table 1).

**Table 1:** Grouping of gallbladder stones combined with occult common bile duct stones.

Risk factor	Training set		Validation set	
	Combined occult CBDS (*n* = 87)	Simple gallbladder stones (*n* = 606)	Combined occult CBDS (*n* = 36)	Simple gallbladder stones (*n* = 259)
Age (years, IQR)	62.3 (54.0–74.0)	51.2 (42–60)	62.1 (53.0–73)	51.0 (43.0–59.0)
Gender (Male/Female, %)	43 (49.4)/44 (50.6)	201 (33.2)/405 (66.8)	16 (44.4)/20 (55.6)	84 (32.4)/175 (67.6)
BMI (kg/m^2^, IQR)	24.3 (22.5–26.2)	24.6 (22.9–26.3)	23.8 (21.9–26.1)	24.3 (22.9–26.2)
Comorbid hypertension (Yes/No, %)	23 (26.4)/64 (73.6)	110 (18.2)/496 (81.8)	3 (8.3)/33 (91.7)	43 (16.6)/216 (83.4)
Comorbid diabetes (Yes/No, %)	8 (9.1)/79 (90.9)	39 (6.4)/567 (93.6)	2 (5.6)/34 (94.4)	15 (5.8)/244 (94.2)
Gallstone size (mm, IQR)	13.7 (7.0–17.0)	12.5 (7.0–15.0)	11.5 (6.0–15.5)	12.7 (8.0–15.0)
Gallstone location (Neck/Non‐neck, %)	13 (14.9)/74 (85.1)	51 (8.4)/555 (91.6)	6 (16.7)/30 (83.3)	29 (11.2)/230 (88.8)
Multiplicity of gallstones (Yes/No, %)	75 (86.2)/12 (13.8)	451 (74.4)/155 (25.6)	31 (86.1)/5 (13.9)	198 (76.4)/61 (23.6)
US‐CBD dilation (Yes/No, %)	28 (32.2)/59 (67.8)	11 (1.8)/595 (98.2)	14 (38.9)/22 (61.1)	4 (1.5)/255 (98.5)
ALT (U/L, IQR)	179.2 (36.0–282.0)	35.3 (14.0–31.0)	145.9 (23.3–132.6)	38.6 (13.0–32.0)
AST (U/L, IQR)	137.4 (27.5–201.5)	29.2 (18.0–27.0)	119.6 (23.6–65.3)	32.2 (18.0–27.0)
ALP (U/L, IQR)	225.9 (100.5–276.0)	82.2 (64.0–93.0)	180.6 (88.8–246.0)	87.4 (65.0–96.5)
GGT (U/L, IQR)	357.3 (101.0–470.0)	44.6 (15.0–41.0)	276.3 (58.0–317.0)	55.2 (15.0–42.5)
TBIL (*μ*mol/L, IQR)	35.9 (12.2–42.0)	14.1 (9.8–16.6)	28.0 (13.2–31.3)	15.0 (9.9–16.6)
DBIL (*μ*mol/L, IQR)	17.3 (2.7–18.4)	2.8 (1.8–3.0)	11.2 (3.0–8.7)	3.2 (1.8–3.2)
HDL (mmol/L, IQR)	1.1 (0.9–1.3)	1.2 (1.0–1.4)	1.2 (1.0–1.3)	1.2 (1.0–1.4)
LDL (mmol/L, IQR)	2.8 (2.2–3.3)	2.9 (2.5–3.3)	2.9 (2.4–3.2)	2.9 (2.5–3.2)
TCHO (mmol/L, IQR)	4.3 (3.5–5.0)	4.6 (4.0–5.1)	4.5 (3.9–4.9)	4.5 (3.9–5.0)
TG (mmol/L, IQR)	1.4 (1.0–1.8)	1.7 (0.9–1.9)	1.3 (0.9–1.7)	1.7 (0.9–1.9)
AMY (U/L, IQR)	93.1 (42.5–79.5)	66.9 (49.0–75.0)	126.7 (50.0–101.2)	67.9 (49.0–77.0)
LDH (U/L, IQR)	221.2 (151.5–234.0)	175.1 (148.0–188.0)	225.6 (154.2–219.0)	180.2 (146.0–194.0)
WBC (10^9^/L, IQR)	7.6 (4.2–7.2)	5.9 (5.1–6.7)	6.6 (4.2–7.2)	6.0 (5.1–6.7)

### 3.3. Univariate Analysis for Gallstones Combined With Occult CBDS

Univariate analysis was conducted following data stratification based on established guidelines and previous studies [14–17]. Chi‐square tests identified significant differences (*p* < 0.05) in Age, Gender, Gallstone multiplicity, US‐CBD dilation, and laboratory parameters (including ALT, AST, ALP, GGT, TBIL, DBIL, WBC, AMY, and LDH) between the two groups. Univariate logistic regression further confirmed gallstone location as an additional statistically significant predictor (*p* < 0.05) (Table 2).

**Table 2:** Univariate analysis of risk factors for gallstones combined with occult CBDS.

Risk factors	Combined occult CBDS (*N* = 87)	Simple gallbladder stones (*N* = 606)	Chi‐square value	Odds ratio	*p* value	*p* value in univariate logistic analysis
Age (years)			40.669	4.292	< 0.001	< 0.001
> 60	49 (56.3%)	140 (23.1%)				
≤ 60	38 (43.7%)	466 (76.9%)				
Gender			8.116	0.508	0.004	0.003
Female	44 (50.6%)	405 (66.8%)				
Male	43 (49.4%)	201 (33.2%)				
BMI			0.072	0.911	0.788	0.698
> 24	55 (63.2%)	396 (65.3%)				
≤ 24	32 (36.8%)	210 (34.7%)				
Gallstone size (mm)			0.863	1.329	0.353	0.285
> 4	22 (25.3%)	123 (20.3%)				
≤ 4	65 (74.7%)	483 (79.7%)				
Gallstone location			3.127	1.912	0.077	0.049
Neck	13 (14.9%)	51 (8.4%)				
Non‐neck	74 (85.1%)	555 (91.6%)				
Multiplicity Of gallstones stones			5.15	2.148	0.023	0.016
Yes	75 (86.2%)	451 (74.4%)				
No	12 (13.8%)	155 (25.6%)				
Comorbid hypertension			2.854	1.620	0.091	0.067
Yes	23 (26.4%)	110 (18.2%)				
No	64 (73.6%)	506 (81.8%)				
Comorbid diabetes			0.532	1.472	0.466	0.339
Yes	8 (9.2%)	39 (6.4%)				
No	79 (90.8%)	567 (93.6%)				
US‐CBD dilation			126.454	25.67	< 0.001	< 0.001
Yes	28 (32.2%)	11 (1.8%)				
No	59 (67.8%)	595 (98.2%)				
ALT (U/L)			164.5	17.679	< 0.001	< 0.001
> 50	61 (70.1%)	71 (11.7%)				
≤ 50	26 (29.9%)	535 (88.3%)				
AST (U/L)			151.515	15.617	< 0.001	< 0.001
> 40	53 (60.9%)	55 (9.1%)				
≤ 40	34 (39.1%)	551 (90.9%)				
ALP (U/L)			221.657	28.296	< 0.001	< 0.001
> 125	59 (67.8%)	42 (6.9%)				
≤ 125	28 (32.2%)	564 (93.1%)				
GGT (U/L)			171.849	21.417	< 0.001	< 0.001
> 60	69 (79.3%)	92 (15.2%)				
≤ 60	18 (20.7%)	514 (84.8%)				
TBIL (*μ*mol/L)			100.236	10.638	< 0.001	< 0.001
> 23.4	39 (44.8%)	43 (7.1%)				
≤ 23.4	48 (55.2%)	563 (92.9%)				
DBIL (*μ*mol/L)			203.459	67.308	< 0.001	< 0.001
> 9.8	35 (40.2%)	6 (1.0%)				
≤ 9.8	52 (59.8%)	600 (99.0%)				
LDL (mmol/L)			0.329	1.546	0.566	0.388
> 4.21	5 (5.7%)	23 (3.8%)				
≤ 4.21	82 (94.3%)	583 (96.2%)				
TCHO (mmol/L)			0.053	0.866	0.818	0.688
> 5.7	10 (11.5%)	79 (13.0%)				
≤ 5.7	77 (88.5%)	527 (87.0%)				
AMY (U/L)			13.231	6.541	< 0.001	< 0.001
> 135	7 (8.0%)	8 (1.3%)				
≤ 135	80 (92.0%)	598 (98.7%)				
LDH (U/L)			42.151	8.414	< 0.001	< 0.001
> 250	17 (19.5%)	17 (2.8%)				
≤ 250	70 (80.5%)	589 (97.2%)				
WBC (10^9^/L)			7.168	2.626	0.007	0.004
> 9.5	13 (14.9%)	38 (6.3%)				
≤ 9.5	74 (85.1%)	568 (93.7%)				

### 3.4. Multivariate Logistic Regression Analysis

Multivariate logistic regression identified Age, ALT, GGT, DBIL, Gallstone location, and US‐CBD dilation as independent predictors of occult choledocholithiasis in patients with gallstones (*p* < 0.05). (Table 3).

**Table 3:** Multivariate logistic analysis of gallbladder stones combined with occult CBDS.

Risk factor	Coefficient	Odds ratio	95% CI	*p* value
Age	0.099	6.473	0.059–0.139	< 0.001
Gender	−0.005	0.890	−0.042–0.032	0.787
Gallstone location	0.058	3.537	−0.001–0.118	0.055
Multiplicity of gallstones	−0.002	1.201	−0.042–0.039	0.936
US‐CBD dilation	0.357	29.048	0.278–0.435	< 0.001
ALT	0.085	3.506	0.009–0.161	0.028
AST	−0.022	0.577	−0.103–0.059	0.595
ALP	0.178	2.633	0.106–0.251	< 0.001
GGT	0.108	6.167	0.050–0.165	< 0.001
TBIL	0.022	1.515	−0.050–0.095	0.548
DBIL	0.334	7.205	0.224–0.444	< 0.001
AMY	0.182	3.537	0.062–0.302	0.003
LDH	0.103	2.915	0.019–0.187	0.016
WBC	0.038	1.625	−0.028–0.104	0.262

### 3.5. Establishment of the Prediction Model for Gallstones Combined With Occult CBDS

The independent risk factors were defined as follows: Age (≤ 60/> 60 years), Gallstone location (non‐neck/neck), US‐CBD dilation (no/yes), ALT (≤ 50/>50 U/L), GGT (≤ 60/> 60 U/L), DBIL (≤ 9.8/> 9.8 *μ*mol/L). The final model was logit(P) = −5.109 + 2.007x1 + 1.175x2 + 3.479x3 + 1.412x4 + 2.199x5 + 2.473x6, where x1–x6 represent Age, Gallstone location, US‐CBD dilation, ALT, GGT, and DBIL, respectively. All variance inflation factors (VIF) were below 2, indicating no significant multicollinearity among the predictors (Table 4).

**Table 4:** Final multivariable logistic regression model for occult choledocholithiasis.

Risk factor	Coefficient	Odds ratio	95% CI	VIF	*p* value
Intercept	−5.109	/	0.002–0.013	/	< 0.001
Age	2.007	7.444	3.629–16.099	1.187	< 0.001
Gallstone location	1.175	3.237	1.223–8.180	1.059	0.015
US‐CBD dilation	3.479	32.435	11.179–102.541	1.117	< 0.001
ALT	1.412	4.103	1.757–9.742	1.579	0.001
GGT	2.199	9.019	3.857–21.929	1.556	< 0.001
DBIL	2.473	11.856	4.072–39.104	1.168	< 0.001

### 3.6. Plotting the ROC Curve and DCA Curve for the Prediction Model

**Figure 1:**
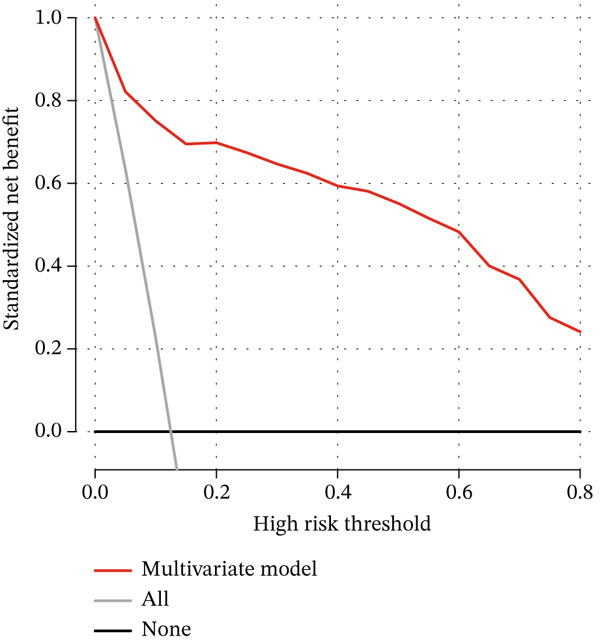
Decision analysis curve for the prediction model of gallstones combined with occult CBDS.

**Figure 2:**
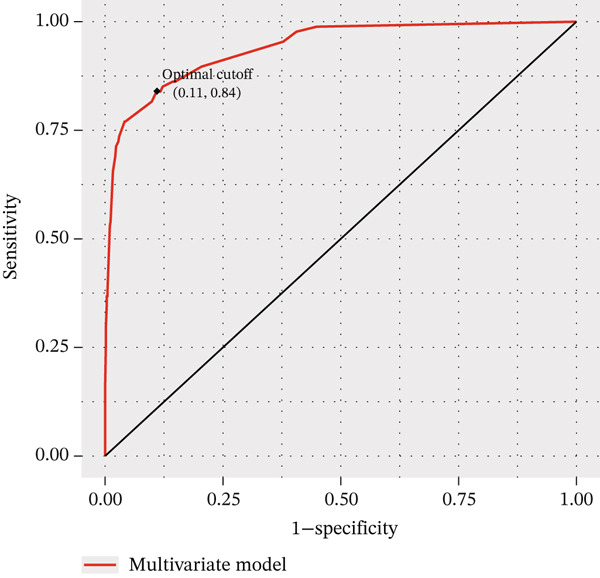
ROC curve for the multifactorial model of gallbladder stones combined with occult CBDS.

The DCA and ROC curves of the model were plotted using *RStudio*. The DCA showed that the multivariable model provided a higher standardized net benefit than the single‐variable model across various risk thresholds. The model exhibited an accuracy of 0.885, a sensitivity of 0.839, a specificity of 0.891, a 95% confidence interval of 0.913, 0.967, and an area under the ROC curve (AUC) of 0.940. (Table 5, Figure 1, Figure 2, Figure 3, and Figure 4).

**Table 5:** Multivariate logistic regression model for gallbladder stones combined with occult CBDS.

Risk factor	Accuracy	Sensitivity	Specificity	95% CI	AUC
Multivariate model	0.885	0.839	0.891	0.913–0.967	0.940
Age	0.743	0.563	0.769	0.611–0.721	0.666
Gallstone location	0.820	0.149	0.916	0.493–0.572	0.533
US‐CBD dilation	0.899	0.322	0.982	0.602–0.702	0.652
ALT	0.860	0.701	0.883	0.742–0.842	0.792
GGT	0.841	0.793	0.848	0.776–0.866	0.821
DBIL	0.916	0.402	0.990	0.644–0.748	0.696

Figure 3Comparison of the multivariate model with DCA for each independent risk factor. (a) DCA comparing the multivariate model with age, gallstone location, and US‐CBD dilation. (b) DCA comparing the multivariate model with ALT, GGT, and DBIL.

**Figure:**
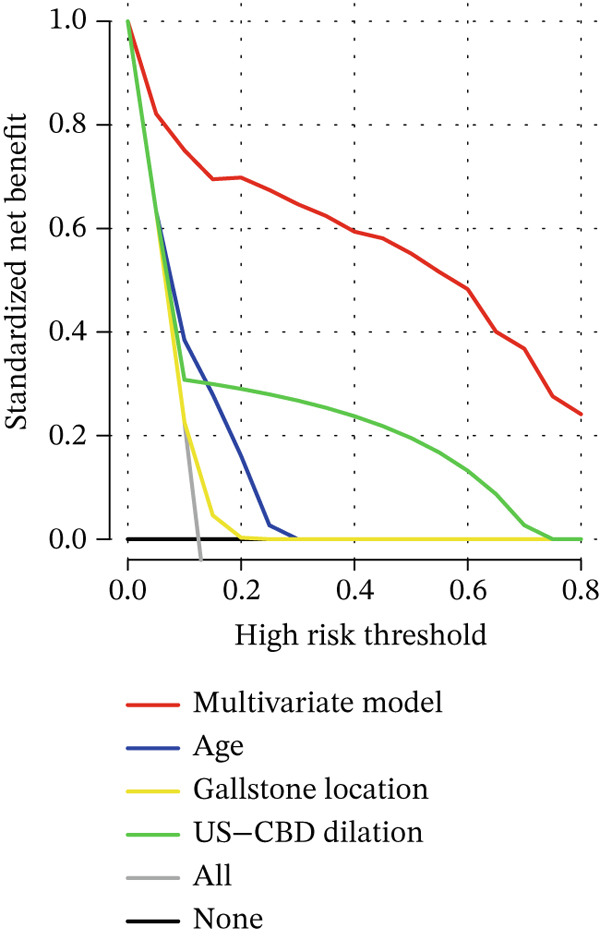
(a)

**Figure:**
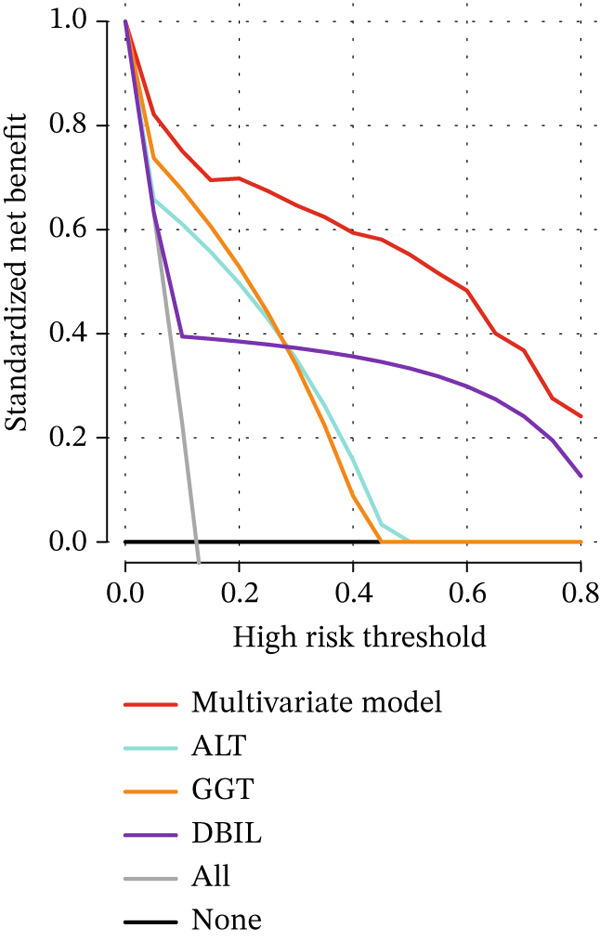
(b)

Figure 4Comparison of the multivariate model with ROC for each independent risk factor. (a) ROC comparing the multivariate model with age, gallstone location, and US‐CBD dilation. (b) ROC comparing the multivariate model with ALT, GGT, and DBIL.

**Figure:**
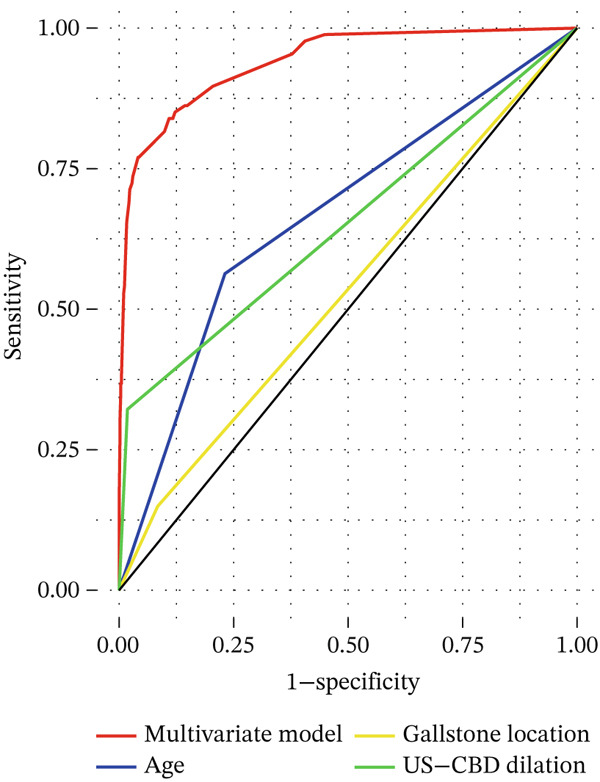
(a)

**Figure:**
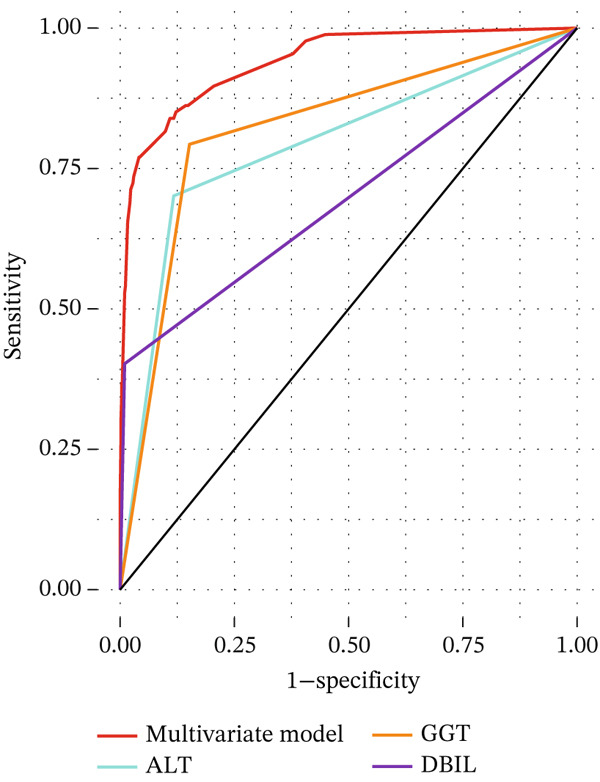
(b)

### 3.7. Bootstrap Resampling and Calibration Curve Plotting

**Figure 5:**
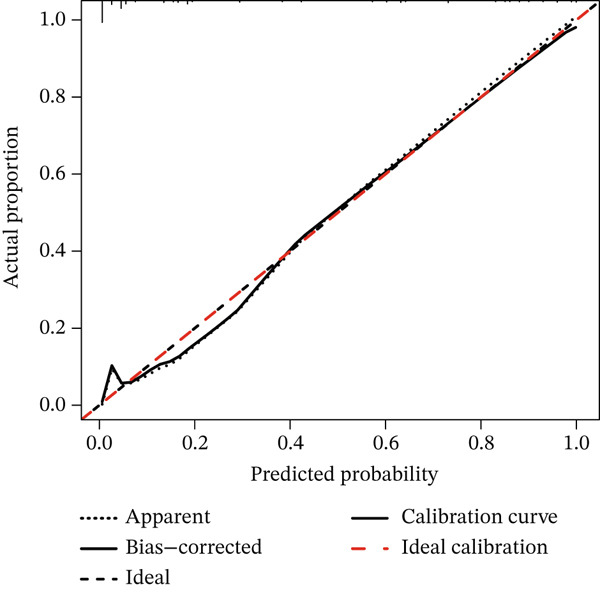
Calibration curve of the predictive model with bootstrap validation (*n* = 2000).

Internal validation via bootstrap resampling (*n* = 2000) confirmed the model’s excellent stability. All predictors demonstrated minimal coefficient variability, with relative bias values below 10% (range: 0.22%–7.28%). The model maintained outstanding discriminative power, showing a bootstrap mean AUC of 0.943 (SD = 0.013) that closely aligned with the original AUC of 0.940 (bias = 0.003; 95% CI: 0.915, 0.967). Furthermore, the calibration curve, plotted to assess the agreement between predicted probabilities and observed outcomes, demonstrated good consistency across the entire probability range. These comprehensive validation findings strongly support the model’s robust performance and clinical applicability. (Table 6, Figure 5).

**Table 6:** Bootstrap validation of the predictive model: coefficient stability and optimism (*n* = 2000).

Variable	Original coefficient	Bootstrap mean	Bias	Relative bias (%)
Intercept	−5.109	−5.275	−0.166	3.25
Age (>60 years)	2.007	2.085	0.078	3.87
Gallstone neck impaction	1.175	1.187	0.012	1.05
US‐CBD dilation (Yes)	3.479	3.618	0.139	3.99
ALT (>50 U/L)	1.412	1.439	0.027	1.94
GGT (>60 U/L)	2.199	2.283	0.084	3.82
DBIL (>9.8 *μ*mol/L)	2.473	2.653	0.180	7.28
Model AUC (95% CI)	0.940 (0.913–0.967)	0.943 (0.915–0.967)	0.003	0.29

### 3.8. Create a Nomogram

**Figure 6:**
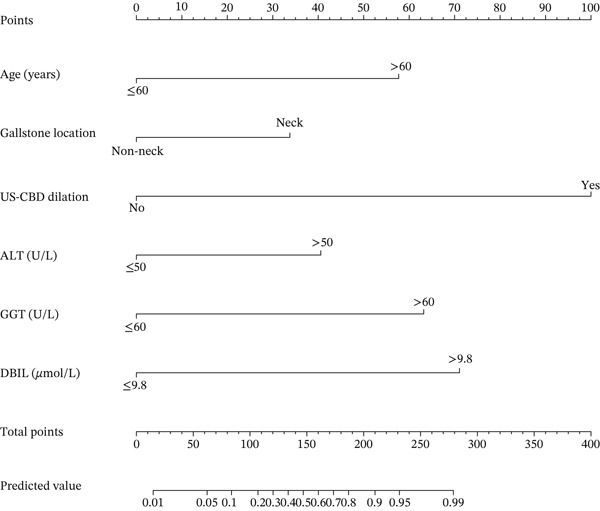
Nomogram for the multifactorial model of gallbladder stones combined with occult CBDS.

Using *RStudio* software, visualize the fitted multifactorial model by creating a nomogram (a graphical representation of a statistical prediction model) (Figure 6).

### 3.9. Model Validation

The model was subsequently applied to the validation set (*n* = 295) to assess its predictive performance. The final results show that the model’s accuracy is 0.871, sensitivity is 0.778, and specificity is 0.884.

## 4. Discussion

Cholecystolithiasis is a common condition in biliary surgery, with most cases detected incidentally during physical examinations via ultrasonography (US). While US demonstrates high sensitivity for gallbladder stones, its diagnostic accuracy for CBDS is limited. In contrast, MRCP exhibits a sensitivity of 89.0% for CBDS detection [18], and EUS reaches 92.9% [19]. However, compared with US, MRCP and EUS require specialized equipment and expertise that may not be available in all medical institutions. Furthermore, EUS is an invasive procedure with specific patient suitability requirements. Therefore, developing a simple and diagnostically valuable screening method for the initial assessment of occult choledocholithiasis is crucial for guiding decisions regarding further diagnostic investigations.

Through univariate and multivariate analyses, this study identified age, ALT, GGT, DBIL, gallstone location, and ultrasonographic common bile duct dilation as independent risk factors for gallstones combined with occult choledocholithiasis. Previous research has suggested that elevated AST, ALT, ALP, TBIL, abnormal pancreatic enzymes, or a common bile duct diameter > 6 mm in patients with acute cholecystitis may indicate concomitant CBDS [20]. Our findings further refine this profile, establishing Age, ALT, GGT, DBIL, gallstone location, and bile duct dilation as core predictive factors.

The incidence of gallstone disease increases with age [1]. Costa et al. [21]. also identified age as an independent risk factor for occult choledocholithiasis. In our cohort, patients with combined occult choledocholithiasis were significantly older than those with simple gallstones (62 years vs. 51 years), and multivariate analysis confirmed age as an independent predictor, indicating a higher probability of occult CBDS in elderly patients with gallstones.

Biliary obstruction can lead to common bile duct dilation [22]. Several studies report that a common bile duct diameter>6 mm increases the likelihood of CBDS [20, 23]. In our study, using US‐indicated common bile duct dilation (diameter>10 mm) as a risk factor, we found an exceptionally high odds ratio (OR) of 32.435, making it one of the strongest predictors. Consequently, preoperative US suggesting common bile duct dilation should raise strong suspicion of concomitant occult choledocholithiasis.

ALT, AST, ALP, and GGT are among the most commonly measured clinical parameters, among which ALT and AST directly reflect liver function [24]. Relevant research has collected data on ALT, TBIL, alkaline phosphatase, and common bile duct diameter in patients with choledocholithiasis to construct a statistical model, with results showing that ALT is one of the independent risk factors for diagnosing choledocholithiasis [25]. Chisholm et al. [20] reported that abnormal elevations in ALT and ALP were associated with CBDS in up to 77.8% of patients with gallstone. Our multivariate analysis confirmed both ALT and GGT as independent risk factors, with ORs of 4.103 and 9.019, respectively. Thus, patients with preoperative elevations in ALT or GGT should be considered for further investigation to rule out occult choledocholithiasis.

DBIL levels directly reflect biliary patency. Multiple studies have shown that DBIL has a sensitivity of 47.1%–79.0% and specificity of 62.7%–86.8% for diagnosing CBDS [26–29]. Our results also confirmed DBIL as an independent risk factor (OR = 11.856). TBIL did not retain significance in the final model, possibly due to the limitations of this single‐center retrospective study and sample characteristics.

This study also identified gallstone location in the neck as an independent risk factor (OR = 3.237). This may be related to the propensity of neck stones to cause functional cystic duct obstruction or facilitate the passage of microliths into the common bile duct; the precise mechanisms warrant further investigation.

The refinement of our model from initial univariate predictors to the final six variables enhances its clinical parsimony. Factors such as ALP, AMY, and LDH, while significant in univariate analysis, did not retain independent predictive value in the multivariate model. This is likely due to collinearity with other stronger predictors (e.g., GGT capturing similar information as ALP) and statistical refinement, which helps prevent overfitting and yields a more efficient tool.

The primary limitations of this study include its single‐center retrospective design, which may introduce selection bias. Although we performed robust internal validation using bootstrap resampling (*n* = 2000), demonstrating good parameter stability (all variables showed relative bias < 10%) and a calibration curve indicating satisfactory agreement between predicted probabilities and observed outcomes, this remains internal validation. Model performance might be optimistically biased due to the single‐institution dataset, and its generalizability across diverse patient populations and different medical centers remains unproven. Therefore, before routine clinical application, the model requires external validation in prospective, multicenter cohorts to confirm its efficacy and robustness.

Despite these limitations, the predictive model developed herein holds clear potential for clinical application. Relying on six readily available preoperative variables, it enables rapid risk assessment. In resource‐limited settings where MRCP or EUS are not routinely accessible, this tool can serve as an effective risk stratification instrument, helping clinicians identify high‐risk patients who would benefit from further biliary imaging (e.g., MRCP), thereby avoiding unnecessary invasive or costly procedures in low‐risk individuals. Furthermore, the model could optimize clinical pathways by facilitating better preoperative planning for high‐risk patients (e.g., considering LCBDE or postoperative ERCP), potentially reducing postoperative complications and the need for reintervention. Future work should focus on multicenter collaborative studies for external validation and explore integrating the model into user‐friendly clinical tools (e.g., web calculators or mobile applications) to enhance its accessibility and impact.

## 5. Conclusion

This study successfully developed and internally validated a logistic regression model for predicting the risk of occult choledocholithiasis in patients with gallstones, based on six easily obtainable preoperative variables: age, ALT, GGT, DBIL, gallstone location, and ultrasonographic common bile duct dilation. The model demonstrated excellent discriminative ability (AUC = 0.940) and good calibration upon internal validation. Although our model shows considerable promise as a practical tool for preoperative decision‐making, its widespread clinical adoption necessitates future external validation. We recommend that subsequent research focus on validating and potentially refining this model using multicenter, prospective data.

## Author Contributions

Ping Zhang and Long‐jiang Chen contributed equally to this work and should be considered co‐first authors. These authors contributed equally.

## Funding

No funding was received for this manuscript.

## Ethics Statement

This retrospective study was reviewed and approved by the Ethics Committee of Wuhu Second People’s Hospital. The requirement for informed consent was waived due to the retrospective nature of the study and the use of anonymized patient data. All procedures were performed in accordance with the ethical standards of the 1964 *Declaration of Helsinki* and its subsequent amendments or comparable ethical guidelines. (IRB Approval Number: 2024‐KY‐017).

## Conflicts of Interest

The authors declare no conflicts of interest.

## Data Availability

The data supporting the findings of this study are available from the corresponding author upon reasonable request.
